# Reviewing reliance on overseas-trained doctors in rural Australia and planning for self-sufficiency: applying 10 years' MABEL evidence

**DOI:** 10.1186/s12960-018-0339-z

**Published:** 2019-01-22

**Authors:** Belinda O’Sullivan, Deborah J. Russell, Matthew R. McGrail, Anthony Scott

**Affiliations:** 1 Monash University School of Rural Health, Office of Research, PO Box 666, Bendigo, VIC 3550 Australia; 2 Flinders University Northern Territory, PO Box 4066, Alice Springs, NT 0871 Australia; 3 University of Queensland, Rural Clinical School, 78 on Canning Street, Rockhampton, Queensland 4700 Australia; 4 Melbourne Institute of Applied Economic and Social Research, The University of Melbourne, Alan Gilbert Building, Parkville, 3010 Australia

## Abstract

**Background:**

The capacity for high-income countries to supply enough locally trained doctors to minimise their reliance on overseas-trained doctors (OTDs) is important for equitable global workforce distribution. However, the ability to achieve self-sufficiency of individual countries is poorly evaluated. This review draws on a decade of research evidence and applies additional stratified analyses from a unique longitudinal medical workforce research program (the Medicine in Australia: Balancing Employment and Life survey (MABEL)) to explore Australia’s rural medical workforce self-sufficiency and inform rural workforce planning. Australia is a country with a strong medical education system and extensive rural workforce policies, including a requirement that newly arrived OTDs work up to 10 years in underserved, mostly rural, communities to access reimbursement for clinical services through Australia’s universal health insurance scheme, called Medicare.

**Findings:**

Despite increases in the number of Australian-trained doctors, more than doubling since the late 1990s, recent locally trained graduates are less likely to work either as general practitioners (GPs) or in rural communities compared to local graduates of the 1970s–1980s. The proportion of OTDs among rural GPs and other medical specialists increases for each cohort of doctors entering the medical workforce since the 1970, peaking for entrants in 2005–2009.

Rural self-sufficiency will be enhanced with policies of selecting rural-origin students, increasing the balance of generalist doctors, enhancing opportunities for remaining in rural areas for training, ensuring sustainable rural working conditions and using innovative service models. However, these policies need to be strongly integrated across the long medical workforce training pathway for successful rural workforce supply and distribution outcomes by locally trained doctors. Meanwhile, OTDs substantially continue to underpin Australia’s rural medical service capacity. The training pathways and social support for OTDs in rural areas is critical given their ongoing contribution to Australia’s rural medical workforce.

**Conclusion:**

It is essential for Australia to monitor its ongoing reliance on OTDs in rural areas and be considerate of the potential impact on global workforce distribution.

## Background: self-sufficiency planning

The capacity for high-income countries to meet their medical workforce needs through education and training of local doctors and thus limit their reliance on overseas-trained doctors (OTDs), called self-sufficiency, remains an important topic of global health workforce policy interest [1, 2]. Ensuring that the geographical distribution of skilled doctors occurs with respect to community health needs of individual countries and sub-regions has important ramifications for achieving the health-related aspects of the United Nations’ Sustainable Development Goals [3, 4]. The World Health Organization’s Voluntary Global Code of Practice on International Recruitment encourages countries to develop human resources data and undertake workforce planning to inform sustainable local human resource development systems that promote self-sufficiency [2, 5]. This is essential to achieve clarity at the national level about actions needed to achieve the right balance of locally trained doctors and reduce the use of trained doctors from low- and middle-income countries, where there is greater relative need, including more prevalent social, environmental and personal risk factors and diseases and fewer health workers [6]. Currently high-income countries benefit from using OTDs to supplement their health service gaps. But, where OTDs are used, the Voluntary Code suggests principles of fairness be applied including countries being transparent about how they are used, supporting these workers, and being mindful of flow on effects to developing countries. Notably, few countries have the necessary national-scale longitudinal workforce data infrastructure and consolidated research evidence for exploring this topic and informing their own nation’s self-sufficiency planning.

Australia is a useful case study of a high-income country that has both a high-quality medical education system and a national policy targeting newly arrived OTDs to work in underserved, mostly rural, communities. As such, exploring the targeted use of OTDs in rural areas provides a useful lens through which to explore self-sufficiency planning nationally. Also, Australia has supported and funded an internationally unique 10-year national longitudinal cohort study of its medical workforce which provides the objective data to inform such medical workforce planning. This is possible if the data area applied to this question and the published research which has been produced from this project is reviewed to make it more accessible for informing this topic.

This research program is known as the MABEL study (which stands for “Medicine in Australia: Balancing Employment and Life”) (http://www.mabel.org.au). It was a competitively funded research project-driven initiative to improve the quality of information about the medical workforce dynamics in Australia and to inform national policy questions. MABEL commenced in 2008 by inviting all 54 750 clinically active doctors in Australia to participate in an annual survey. The sampling frame was an Australian Medical Publishing Company (AMPCo) population listing with updated details of doctors’ contact information for regular mailing purposes (the best available directory at the time). To facilitate response from doctors in remote communities (important for informing key policy questions), pre-paid monetary incentives, not conditional on response ($AU100), were applied. In the first year, 19.4% of Australia doctors responded in hard copy or via the secure online survey: 3906 general practitioners (GPs) and GP registrars (undertaking postgraduate training in general practice), 4596 other (non-GP) specialists (called specialists hereafter), 1072 other (non-GP) specialists (undertaking postgraduate training in a non-GP specialty), and 924 hospital non-specialists.

Respondents were assessed as being relatively representative of all Australian doctors, although compared to the total AMPCo population, the respondents had slight over-representation of (no more than 4%) of age groups less than 60 years, females (6%) and non-GP specialists (5%) [7]. After geo-coding responses, doctors in different remote areas were also slightly over-represented (< 2%), possibly related to the pre-paid incentive [7]. In each year since 2008, initial respondents are re-surveyed, with top-up from new graduates or new doctors arriving in Australia [8]. The survey is sent to both Australian-trained (including New Zealand, as per Australia’s policies) and OTDs, (completed medical school training in any other country). Around 16% of respondents were OTDs (*n* = 1 719), including 21% of GPs (*n* = 805), but the representativeness of these respondents was not assessed as there was no population data to base this on.

Each survey comprises around 100 questions covering the doctor’s characteristics, specialty, work location and practice, workload, job satisfaction and remuneration. The annual operational costs (excluding research production) are $350 000 Australian dollars (Aus$), approximating $273 288 United States dollars (US$) (exchange rate March 2018). Now with around 80 000 responses from 20 000 different doctors, completed over 2008–2015, it comprises the most comprehensive source of national workforce evidence for informing planning for self-sufficiency in Australia because it covers doctor type, training and location as well as a range of other rich covariates. However, the evidence generated from the project has never been synthesised to explore the topic of rural self-sufficiency.

### Australian context

Rural and remote areas, where around 30% of the Australian population and 65% of Indigenous Australians reside, have substantial and ongoing difficulties related to the recruitment, retention and overall supply of skilled doctors who work at the broad scope to cover the bulk of community needs [9, 10] (Table 1). These issues occur within the context of higher needs of rural populations for medical services: rural and remote residents have, on average, lower incomes, more health risk factors, poorer access to local health services and a higher burden of illness than metropolitan residents [11, 12].

**Table 1:** Population distribution and supply of doctors per 100 000 population, by remoteness, 2015 [11, 61, 62]

	Major cities	Inner regional	Outer regional	Remote/very remote	Whole country
Area (1000 km squared)	19	246	784	6 639	7688
Number of people (,000)	16 864	4303	2085	525	23 778
% Indigenous population^a^	34.8	22.0	21.8	21.4	100
% non-Indigenous population	71.3	18.3	8.7	1.7	100
General practitioner full *time equivalents* per 100 000 population^b^	111.6	113.8	116.3	135.5	113.9
General practitioner full *service equivalents* per 100 000 population^c^	99.4	98.4	88.5	69.5/55.1	95.1
Other medical specialist full *time equivalents*^b^	162.1	82.7	61.5	34.2	134.3

Location is based on location of main place of work and categorised according to remoteness using the 5-level Australian Statistical Geography Standard Remoteness Area (ASGS-RA) classification [63]

^a^Total indigenous population = 669 900; 3% of the total Australian population

^b^Full *time equivalents* (of general practitioners (GPs), other specialists) per 100 000 population: based on total hours worked in the past week as collected in the Australian Health Practitioner Regulation Agency annual medical workforce survey. This measure poorly accounts for high workforce turnover, use of locums, OTDs and poorer population health status

^c^Full *service equivalents* of GPs per 100 000 population: approximation of hours worked, based on Medicare Benefits Schedule billing (universal billing system) data of number of days worked, volume of services and schedule fees. This measure poorly accounts for factors listed above and additionally salaried activity (not remunerated by Medicare Benefits Schedule billing) which is more common practice by GPs based in small rural and remote areas

A range of solutions have been introduced to improve access to medical services in remote areas, including through outreach primary care and retrieval services such as the Royal Flying Doctor’s Services, which commenced in 1928 and continue [13]. However with around a third of Australians based in rural areas and working in rural industries of central importance to the nation’s Gross Domestic Product, resident health professionals providing comprehensive care is considered more optimal for achieving health outcomes. A key complexity for policy is that Australia is a large country with a large number of communities to serve, vast distances between communities and large sections of arid uninhabited land.

National rural health workforce policies rapidly emerged from the Australian government, from the late 1990s to stimulate rural workforce supply from locally trained students. These included increasing medical school places (1300 in 2004 to 3300 in 2016) to boost overall medical workforce capacity with the potential to have some impact on rural supply through market effects. Within enrolment numbers, medical courses were required to enrol a minimum of 25% rural-origin medical students, provide at least 1 year’s rural immersion for 25% of students, bond 25% of enrolled students to work in a district of workforce shortage (typically rural) after completing the medical course for a pre-set period of time, and additionally half of all GP training was located in rural areas [9, 14]. Further, regulatory policies emerged and national legislation passed in 1997 restricted newly arriving OTDs to work in a district of workforce shortage for between 3 (more remote) and 10 years (least remote areas) and to access a provider number which is required to get reimbursed for private clinical services via Australia’s universal health insurance scheme, Medicare (through which most doctors are reliant unless working in public hospitals) [15].

The principle of self-sufficiency was espoused by Australia’s government in a 2004 national health workforce strategy [16]. It was broadly understood as producing sufficient locally trained doctors to minimise the use of OTD, with OTDs still having a role in supplementing the domestic skills market [2, 16–18]. No clear benchmarks were set.

Similar to the United Kingdom, United States and Canada, around one quarter of Australia’s doctors are trained in medical schools based in other countries [19]. Australia’s immigration department approved 2820 temporary resident visas (class 457 and 442/402) for OTDs in 2014–2015 [20]. Around half of the OTDs entering Australia in the decade between 2005 and 2006 and 2014 and 2015 were from low- or middle-income countries (53% of those on temporary and 49% on permanent visas), though little is known about changes in the country of origin of OTDs over time [21, 22].

While the degree of under- or over-supply of the broader medical workforce has been regularly debated and analysed in national reports [23–26], the discussion has mainly focused on macro-level stocks and flows and has not included any discussion about rural areas specifically. National data provides some conflicting evidence about the comparative overall supply of doctors in rural versus metropolitan areas, based on different measures and methods used [14] (Table 1). However, these national data are not differentiated enough by doctor type, country of training and doctor career stage and lack many covariates of interest, so they do little to inform the level of self-sufficiency and how to proceed as a nation. Meanwhile, longstanding longitudinal evidence from the MABEL project has strong application to this question.

This review draws on over 10 years’ evidence and applies additional stratified analyses of the MABEL longitudinal data to inform planning for self-sufficiency of the rural medical workforce in Australia. It explores the (1) patterns in rural work location by overseas- and locally trained doctors, differentiated by specialty and career stage, and (2) factors associated with rural work outcomes by locally trained doctors, and (3) discusses the implications of this evidence in light of the international literature.

## Findings from MABEL

### Rural work location patterns by country of basic medical training

**Fig. 1:**
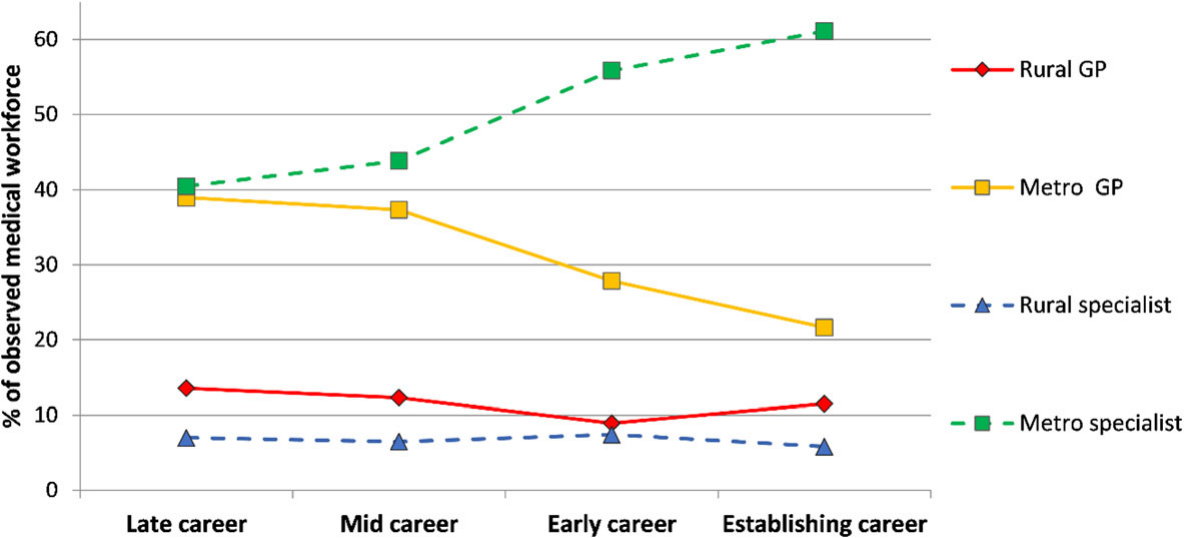
Types of locally trained graduate doctors and their 2008–2013 work location, by when they graduated and entered the medical workforce in Australia. The methods used for this figure have been published elsewhere [27]. Based on locally trained respondents answering the MABEL question “in what year did you complete your basic medical degree”, four groups were categorised (1) late career = graduated during 1970s (mostly aged 55–70 years at time of survey); (2) mid-career = graduated during 1980s (mostly aged 45–60 years); (3) early career = graduated during 1990s (mostly aged 35–50 years); (4) establishing career = graduated during 2000s (mostly aged 25–40 years). Work location was identified by a question “where is your main place of work – town and postcode”, geocoded and categorised rural or metropolitan based on the Modified Monash Model. Each doctor responded to between one and six surveys, thus contributing up to six aggregate person-years of work location data 2008–2013. Non-response weights were applied. Respondents for each cohort were as follows: 1970s = 18%, 1980s = 23%, 1990s = 22%, 2000s = 37% [64]

The MABEL data, applied as a new stratified analyses, shows the geographical distribution (by 2008–2013 work location) and specialty type of the locally trained medical workforce according to the year that they graduated from medical school and entered the medical workforce (Fig. 1) [27]. Figure 1 indicates that smaller proportions of locally trained graduates entering the workforce more recently (since 2000) were working as GPs compared to graduates from the 1970s and 1980s; a higher proportion are pursuing non-GP specialist careers. And of the locally trained students who entered the workforce in the 1970s and 1980s, a higher proportion were working in rural areas compared with graduates from the 1990s and 2000s.

MABEL data, again applied as a new stratified analyses in Table 2, shows the distribution of the current locally trained and OTD workforce by specialty type and work location (large regional or smaller rural communities) (respondents to the MABEL survey 2008–2013) according to the year that they entered the medical workforce. Table 2 indicates that OTDs comprise proportionally less GPs and other medical specialists of entrants to the Australian medical workforce in the 1970s, working in both large regional (12% and 17% respectively) and smaller rural and remote areas (16% and 11% respectively). However, OTDs comprise the majority of rural GPs and other specialists among those entering the workforce in the most recent period (2005–2009) in both large regional (59% and 60% respectively) and smaller rural and remote areas (67% and 66% respectively). The same pattern exists for the metropolitan distribution by cohort although with a less pronounced effect by cohort than is observed for rural locations. This pattern is relatively consistent across the period in the 1990s when regulatory policies required OTDs to work in districts of workforce shortages (mainly rural areas) and when more rurally oriented rural workforce pipeline policies were introduced into Australia.

**Table 2:** Location of main place of work when did the MABEL survey (2008–2013) for locally trained and overseas-trained doctors (OTDs) by the period they entered Australian medical workforce

		General practitioners			Other medical specialists		
Period entered medical workforce	Category	Metropolitan (%)	Large regional or rural (> 15 000 population) (%)	Small rural and remote (< 15 000 population) (%)	Metropolitan	Large regional (> 50 000 population) (%)	Small regional or remote (< 50 000 population) (%)
1970s	Locally trained	84.2	87.6	83.6	86.8	82.7	88.6
	OTD	15.8	12.4	16.4	13.2	17.3	11.4
1980s	Locally trained	84.3	81.4	82.1	86.2	82.6	79.7
	OTD	15.7	18.6	17.9	13.8	17.4	20.3
1990–1994	Locally trained	72.3	60.9	68.1	80.7	71.2	68.5
	OTD	27.7	39.1	31.9	19.3	28.8	31.5
1995–1999	Locally trained	79.4	63.1	52.9	73.6	67.3	62.6
	OTD	20.6	36.9	47.1	26.4	32.7	37.4
2000–2004	Locally trained	72.3	50.6	52.0	74.7	51.2	44.0
	OTD	27.7	49.4	48.0	25.3	48.8	56.0
2005–2009	Locally trained	54.4	41.0	33.3	66.5	40.1	34.1
	OTD	45.6	59.	66.7	33.5	59.9	65.9

“Period entered medical workforce” is based on a question in the MABEL survey “in what year did you complete your basic medical degree”. From this, the first year the doctor (general practitioners, other medical specialists, or registrars undertaking formal training for general practice or other specialties) entered the Australian medical workforce was calculated. For OTDs, this is the year that OTDs first registered and commenced work in Australia. OTDs are identified as having completed their basic medical degree in a country other than Australia. Data are unweighted to avoid sample bias related to different age and sex distribution of OTDs relative to locally trained doctors. The methods applied to this analysis have been published elsewhere [27]. “Location of main place of work” was identified by a question “where is your main place of work – town and postcode?”, geocoded and categorised large regional or rural, small rural or metropolitan based on the Modified Monash Model [64]. Each doctors responded to between one and six MABEL surveys, thus contributing up to six aggregate person-years of work location data 2008–2013. The different geographic categorisation applied to specialists reflects they have larger population catchment and practice infrastructure requirements [29]

Other published evidence from the MABEL study shows that OTDs are over-represented among rural GPs: they comprise 27% of all GPs, but only 22% of GPs in metropolitan and regional centres, and 36–38% of GPs in towns of < 50 000 population [28]. Furthermore, high proportions (around 60–70%) of rural OTDs working as GPs are restricted as to where they can practice (under the Australian policy requirements) [28]. Similarly, OTDs comprise 27% and 29% of other medical specialists in metropolitan and large regional centres of > 50 000 population respectively, and 38% of specialists in smaller regional centres of < 50 000 population [29].

### Broad factors related to rural work location of locally trained doctors

To achieve self-sufficiency, it is critical to identify the drivers of locally trained doctors working in rural areas, so that the locally trained medical workforce is sufficiently well-distributed to meet community needs. A number of factors are globally recognised as pre-requisites for building rural workforce capacity overall [10, 30]. However, by reviewing the published evidence from the national longitudinal MABEL study, it is possible to clarify the mix of impact factors with respect to the Australian context. This is particularly important given the current increased production of locally trained doctors is not yet translating to more production of GPs and better rural distribution.

The published evidence from the MABEL project identifies that GPs who spend at least 6 years of their childhood in a rural location (and for other medical specialists, at least 11 years) are more than twice as likely as metropolitan-origin doctors to work in rural areas [31]. Additionally, vocational training for general practice in rural areas is associated with continuing to work rurally for at least 5 years after vocational registration, with the effect being stronger for doctors with a childhood rural-origin [32].

GPs who are principals or associates of the practice are retained for longer in the current practice than contracted or salaried GPs [33], with the latter having significantly higher mobility rates [34]. GPs with additional skills in anaesthetics, obstetrics and emergency medicine are more likely to be working in smaller communities (15–50 000 population) compared with GPs without these skills [35]. GPs involved in hospital or procedural work are also likely to stay in rural practice for longer [33]. Of other medical specialists, general physicians and general surgeons are more likely to work in smaller regional centres (< 50 000 population), relative to anaesthetists [29].

Rural GPs and other medical specialists have higher average weekly working hours, more on-call responsibilities and less opportunities for continuing professional development, although they report equivalent job satisfaction as their metropolitan counterparts [29, 36, 37]. Financial incentives are unlikely to mobilise GPs to change work locations; 65% report they would not move irrespective of the package proposed. The average annual incentive needed to compensate doctors for the least favourable work conditions (a town with 1-in-2 on-call requirements and a smaller inland community where getting locums is difficult regardless of financial incentives) is $237 000 ($Aus, as of 2012) (approximating 184 933 United States dollars (US$) as of exchange rate 7 March 2018) [38]. Locum support is the strongest factor that GPs’ report would influence their ongoing retention in rural areas [39].

The supply and retention of doctors in rural areas is also affected by a range of community and demographic factors. GPs stay longer in rural communities with more than 10 000 people than in smaller rural or remote communities [33]. GP mobility rates increase with increasing geographical remoteness—5% of regionally located GPs compared with up to 18% of those based in very remote areas move to a different town or city each year [34]. Additionally, mobility is higher for younger GPs and GPs who recently moved to a rural location [34]. Male GPs with children in secondary school are less likely to work in any-sized rural town compared to male GPs who have primary school-aged children. Female GPs with a spouse/partner in the workforce are less likely to work in smaller communities (< 15 000 population) compared with those non-partnered or with a partner not in the workforce [40].

Around 19% of medical specialists participate in outreach work, regularly travelling away from the main practice to service a rural community [41]. Such outreach services are to larger regional centres (58%) and more outlying regions and remote communities (42%) [42]. Fifty-two percent of specialists doing outreach work sustain their services to the same community for at least 3 years [43]. In Australia, a national policy subsidises travel costs for 20% of all specialist outreach workers. Those supported by this are more likely to be specialists in fields related to national rural health priority areas and to visit more remote locations [44, 45].

### Implications for self-sufficiency planning

Several implications for self-sufficiency planning are notable from applying the new stratified analyses of MABEL data and the review of a decade of published evidence from this project. Firstly, Australia relies heavily on OTDs for rural medical workforce capacity, both as GPs and other medical specialists, for access to medical services in large and small communities and this reliance is increasing. Recent locally trained graduates are increasingly choosing specialties other than general practice, reducing their likelihood of working in rural areas where the broader specialist and sub-specialist infrastructure is more limited. It is imperative for self-sufficiency that a higher proportion of locally trained doctors chooses general practice and other generalist specialties. Altman et al. noted defining and professionally recognising generalist medicine can help students see it as something concrete to be an expert in [46]. Additionally, a National Rural Generalist Training Pathway is currently under development in Australia to strengthen opportunities for local students training in rural medical schools, to continue to work in rural regions in pre-vocational and vocational training, developing skills in all the areas of medical practice relevant for rural and remote communities and connecting to rural communities in the process [47].

Secondly, increasing self-sufficiency demands sustained investment for locally growing rural doctors. In this area, the published MABEL evidence denotes and quantifies the drivers of rural supply and distribution for doctors in Australia, which concurs with international evidence: from enrolment of rural origin students; providing consecutive rural training opportunities during undergraduate, pre-vocational and vocational training; and developing attractive advanced skills training and generalist career pathways [10, 30, 48–50]. Australia already has a number of rural selection and undergraduate training policies, but they are not producing the size and quality of medical workforce needed in rural Australia partly because there is limited continuity in prevocational and vocational rural training. New Australian policy initiatives like the Rural Primary Care Stream will provide funding for educational support for junior doctors (from their first year post-graduation to fifth year) working and training in rural primary care settings and the Integrated Rural Training Pipeline including new Regional Training Hubs, aiming to stimulate the development of the right balance of generalist and more specialist doctors for rural areas via more continuous post-graduate rural training [51]. However, many of these policies are only in their early stages of implementation, and achieving successful outcomes from these relies on extensive policy consensus and genuine effort across the various levels of government, as well as engagement and real action within regional health and hospital services, general practices, regional educational units and regional communities. Rural workforce outcomes from educational interventions can have long lag times of 10 to 15 years, which span several election cycles, demand long-term vision and motivated system-level leaders. Improved outcomes for rural communities are more likely where there is accountability of all stakeholders involved in the long rural medical training pathway, for the rural workforce outcomes communities want. Also, incentives and formal agreements may be needed for different stakeholders to collaborate. Finally, rural communities should be involved in developing such policies that are meant to serve them.

There are still some important gaps in Australian rural workforce policy notable based on the rural workforce drivers identified from the review of the MABEL evidence, mainly related to improving sustainable working conditions for rural doctors [14]. Innovations in health service employment structures are acutely needed to better manage onerous on-call requirements, create feasible rosters, improve access to locums and increase access to professional support. To a large extent, rural working conditions can be improved by increasing the critical mass of a complementary range of doctors working in healthcare teams, at broad scope. With respect to building critical mass, there is great potential to invest more in supporting existing rural doctors, including OTDs, to build additional complementary skills that allow them to contribute to the service demands across rural and remote communities.

Finally, high-quality regional workforce planning is sorely needed for Australia to achieve self-sufficiency and minimise reliance on OTDs. Regional planning firstly requires a degree of societal determination to transition away from continued heavy OTD use and develop of local training pathways as the alternative. Research from the United States suggests that the transition away from OTDs at the community level is complicated and takes time [52]. Though, at a policy level, OTD withdrawal can be enacted suddenly, with potentially detrimental effects on immediate access to medical care in communities that are more OTD dependent. Although some communities have strong potential to “grow their own”, other rural communities still have limited links with locally trained/training doctors and restricted chances of attracting them. Even for communities instituting more training pathways, it takes time for the locally trained workforce capacity to grow and differentiate to sufficient levels to meet community need. As such, continued targeted OTD supply and ongoing support remains a critical issue in Australia.

A 2012 Parliamentary Inquiry identified that OTDs have poor access to orientation programs, professional registration, career opportunities and social supports [53]. This is somewhat being addressed with a current drive to encourage more OTDs to pursue equivalent educational standards as Australian-trained doctors in vocational training, but education programs alone will likely not be enough [54]. Support for addressing the broader professional and personal (including social) factors enabling OTDs who are restricted by where they can work to enjoy rural practice and rural living is also needed [28]. Many OTDs begin working in Australia in rural and remote settings under some of the most isolated conditions, treating people with the most complex health problems, including working in Indigenous populations who need culturally safe care. This happens before any equivalence of training is achieved and with no working knowledge of the Australia health system. Allowing more OTDs to acclimatise to the Australian system before requiring rural service terms and providing regular cultural awareness training would be a good investment.

It is incumbent on Australia as party to the Global Code of Practice on International Recruitment, to provide structured support for OTDs, whilst also monitoring the degree of reliance on OTDs and the potential impact on global workforce distribution [55]. Notably, this relies on good longitudinal evidence differentiating types of health workers (locally or overseas trained) and practice patterns which is regularly and transparently published. Workforce data as a resource needs to be sustainably funded in all countries for objectively informing both national and global health workforce planning policies [56–58].

### Limitations

The scope of this paper was confined to summarising available evidence from the MABEL research program, which limited the aspects of rural self-sufficiency investigated as such. It did not explore the level of reliance on OTDs to complement the range of skills across the broader workforce including in areas like psychiatry, where there are not enough locally trained psychiatrists for increasing population needs [59]. Also, the current report did not differentiate the rural work patterns by locally trained international students or local rural bonded students, which is planned as part of ongoing MABEL research. In terms of global impact of Australia’s use of OTDs, this paper also did not explore implications for the country of OTD supply, but all nations and particularly low- and middle-income countries are disadvantaged through out-migration, given the investment they put into training health workers for their country [60]. Where possible, there might be the potential for bilateral and multi-lateral agreements between professional bodies, nations, and regions regarding using OTDs, with respect to both training and practice and support. The evidence produced may not readily translate to other countries which have different self-sufficiency challenges, health system structures, rural workforce policies and medical service providers. Nevertheless, this work may provide an example of an attempt to consolidate the best available national evidence for informing national self-sufficiency planning.

## Conclusions

This report draws on evidence which identifies patterns of work by overseas- and locally trained doctors in Australia, to inform planning of self-sufficiency of the rural medical workforce in Australia. The evidence identifies that Australia relies heavily on OTDs working as GPs and other medical specialists in large and small rural communities, with new OTDs supplementing the lower proportion of recent locally trained doctors in rural areas. Increasing the locally trained generalist rural workforce requires sustained policies, enrolling rural-origin students into medicine, providing ongoing rural training opportunities, promoting generalist medical careers and building sustainable rural working conditions. Meanwhile, OTDs are essential to rural workforce capacity and their support should be incorporated as a key part of ongoing rural medical workforce planning. Australia, like all similar high-income countries, should monitor the reliance on OTDs within rural medical services and be acutely mindful of the potential impact on global workforce distribution.
